# Can ChatGPT understand genetics?

**DOI:** 10.1038/s41431-023-01419-4

**Published:** 2023-07-05

**Authors:** Frank Emmert-Streib

**Affiliations:** Predictive Society and Data Analytics Lab, Faculty of Information Technology and Communication Sciences, Tampere University, Tampere, Finland

**Keywords:** Medical genetics, Genomics, Genetics research, Data processing

In November 2022, OpenAI released an online chatbot called ChatGPT which is a question-answering system based on deep learning. The suffix “GPT” means generative pre-trained transformer [1] that is a type of large language model (LLM) consisting of billions of parameters trained via transfer learning [2]. This makes such LLMs highly complex and the optimization of the model’s parameters a very difficult task. Yet the usage of ChatGPT is very easy because by typing a question in a natural language like English, ChatGPT generates an answer in English. While this is intriguing, ChatGPT is a multi-purpose tool and the question is what to do with it? More precisely, can ChatGPT be used in a particular application area? In [3], Duong and Solomon studied an application of the chatbot in genetics by testing its expertise.

The reason why this needs to be studies is that when training ChatGPT, very large corpora of text about essentially all conceivable topics are used including to some extend also publications about biomedical, medical and clinical research utilizing genetics and genomics. However, ChatGPT is not designed to focus on either of these nor on any other area but it is a generic tool. This implies that, currently, the expert level of this tool in particular application domains is largely unknown which means that in some areas ChatGPT could be “competent” while in others it could fail or it could be even a polymath. In order to gain insights into the expertise of ChatGPT about genetics, in [3] the answers to multiple-choice questions were compared to human generated answers gathers from social medial platforms. This creative usage allowed to show that in comparison to a heterogeneous group of humans with an uncontrolled background, ChatGPT performed similarly with an overall accuracy of almost 70%.

To gain a better understanding of these results, we show two of these questions; see Supplementary Information [3]. Question (398): A patient is suspected to have a genetic cause of rhabdomyolysis. A condition affecting which pathway or mechanism would be most likely?

Possible answers:

1. Bile acid synthesis
2. Fatty acid oxidation
3. Peroxisomal metabolism
4. Urea cycle

Question (393): You follow a young adult who has been diagnosed with Lynch syndrome. Her father is also affected; he was diagnosed with colorectal cancer in his late 30s. According to current guidelines, at about what age is colonoscopy recommended for your patient?

Possible answers:

1. 10–15 years of age
2. 20–25 years of age
3. 30–35 years of age
4. Around 50 years of age

These examples demonstrate that the asked questions were far from simple. As a side note, we would like to remark that achieving an accuracy of around 66% for the human (control) group of social media users allowed Duong and Solomon to infer that these individuals possess a good educational background on the subject. This is because a layperson would likely only be able to guess the answers to such questions. Hence, for ChatGPT to perform similar to this group is remarkable.

Having established that ChatGPT has a certain amount of expert knowledge about genetics the next question is: For what can it be used? One purpose that comes immediately to mind is as a tutoring system for learning genetics. A big advantage compared to human tutors or even teachers is the availability 24/7 and tireless patience over arbitrary long learning sessions. However, considering the performance of ChatGPT with an accuracy around 70% it should be noted that this tutor is still fallible and there is room for improvement. Of course, also human tutors do not know everything and for this reason we limit this risk by requiring educational degrees on various levels, e.g., high school, college, university etc., before teachers are permitted to educate pupils or students. Similarly, one could either have several specialized versions of ChatGPT or one generic version but in either case with a “graduation certificate”. Certainly, even in its current form ChatGPT can be used for this purpose but the learner requires a high level of maturity by not taking every answer as ground-truth but as a suggestion that needs to be checked. Hence, if used in this way ChatGPT is already now a good support system for learning.

Another more intricate usage of ChatGPT would be for research in genetics and genomics. Leaving biomedical text mining problems like named entity recognition (NER) and relation detection (RD) aside, for which it can certainly be used [4], at the moment it remains unclear if ChatGPT is useful in this context and for what particular problem. For this reason, we asked ChatGPT. In the following, we show two selected answers from a longer list of possible suggestions that were reasonable but too unspecific:

- Experimental design: ChatGPT can assist in designing experiments by providing guidance on sample sizes, control groups, statistical analysis, and selecting appropriate techniques for genetic studies.
- Hypothesis generation: Researchers can use ChatGPT to generate novel hypotheses based on existing data or theories. By feeding the model with relevant genetic information, it can suggest potential connections or mechanisms that researchers can further investigate.

While the first suggestion is related to tutoring discussed above, the generation of hypothesis sounds revolutionary. However, one problem with this is mentioned in the suggestion itself which is that, currently, it is not possible to upload external files as input for ChatGPT containing either experimental data or text data for fine-tuning the model to a particular problem. This is a severe limitation because it make the creation of problem-specific hypothesis more challenging or even impossible. Still, overcoming this restriction seems very feasible in future releases of ChatGPT allowing such updated features. Only then an analysis of this could be conducted to see if this is indeed possible.

Overall, there are many more open questions that need to be considered [5] but the study by Duong and Solomon [3] gave an interesting particular example for the application of ChatGPT in genetics which should be even transferable to other fields as well. At this point, it is hard to foresee all possible applications of ChatGPT or similar conversational AI systems. However, we have no doubt that ChatGPT is here to stay. Curiously, the only way to explore its capabilities and limitations seems old fashioned human thinking.
